# Assessment of turbulent blood flow and wall shear stress in aortic coarctation using image-based simulations

**DOI:** 10.1186/s12938-021-00921-4

**Published:** 2021-08-21

**Authors:** Romana Perinajová, Joe F. Juffermans, Jonhatan Lorenzo Mercado, Jean-Paul Aben, Leon Ledoux, Jos J. M. Westenberg, Hildo J. Lamb, Saša Kenjereš

**Affiliations:** 1 Department of Chemical Engineering, Faculty of Applied Sciences, Delft University of Technology, Delft, The Netherlands; 2 J.M. Burgerscentrum Research School for Fluid Mechanics, Delft, The Netherlands; 3 Department of Radiology, Leiden University Medical Center, Leiden, The Netherlands; 4 Pie Medical Imaging BV, Maastricht, The Netherlands

**Keywords:** Magnetic resonance imaging, Computational fluid dynamics, Turbulence, Aorta, Coarctation, Phantom

## Abstract

In this study, we analyzed turbulent flows through a phantom (a 180$^{\circ }$ bend with narrowing) at peak systole and a patient-specific coarctation of the aorta (CoA), with a pulsating flow, using magnetic resonance imaging (MRI) and computational fluid dynamics (CFD). For MRI, a 4D-flow MRI is performed using a 3T scanner. For CFD, the standard $k-\epsilon$, shear stress transport $k-\omega$, and Reynolds stress (RSM) models are applied. A good agreement between measured and simulated velocity is obtained for the phantom, especially for CFD with RSM. The wall shear stress (WSS) shows significant differences between CFD and MRI in absolute values, due to the limited near-wall resolution of MRI. However, normalized WSS shows qualitatively very similar distributions of the local values between MRI and CFD. Finally, a direct comparison between in vivo 4D-flow MRI and CFD with the RSM turbulence model is performed in the CoA. MRI can properly identify regions with locally elevated or suppressed WSS. If the exact values of the WSS are necessary, CFD is the preferred method. For future applications, we recommend the use of the combined MRI/CFD method for analysis and evaluation of the local flow patterns and WSS in the aorta.

## Background

Coarctation of aorta (CoA) is a congenital condition in which the aorta has a narrowing, usually in the thoracic descending aorta distal to the branching arteries of the aortic arch. The narrowing of the artery causes flow acceleration, where a turbulent-like flow may occur during the systolic phase [1]. It has been shown that the transitional and turbulent flow in CoA leads to aberrant blood flow in the narrowing and a vortex-like recirculation pattern distal to the stenosis [2]. Due to the stenosis and onset of turbulence, the wall shear stress (WSS) is also elevated, and the presence of turbulence may cause oscillations of its values [3]. This type of flow may cause, among others, degradation of the arterial wall, initialization of an aneurysm, and atherosclerosis [4].

Several studies have assessed the hemodynamics of this pathology using magnetic resonance imaging (MRI) [5, 6]. However, due to the relatively low spatial resolution of MRI, the flow velocity in the proximity of the wall and its derived quantities such as WSS may be incorrect [7]. Several recent studies have identified image-based computational fluid dynamics (CFD) to be a good alternative to study blood flow in CoA [8, 9]. However, these previous studies have not addressed the important effects of locally generated turbulence in CoA, as demonstrated by Gaze et. al. [10]. The turbulent flow in CoA has been simulated by using various turbulence modeling approaches: (i) the Reynolds-averaged Navier–Stokes (RANS) ([11]; (ii) large-eddy simulation (LES) [12]; and finally, (iii) direct numerical simulation (DNS) [13] methods. The DNS and LES proved to perform very well for transient and turbulent flow regimes for arteries with stenotic regions [14]. However, because of the huge computational costs associated with high temporal and spatial resolution requirements of LES and DNS, these approaches are less suitable for clinical applications [15]. To meet demands on a computationally efficient and sufficiently accurate CFD approach, we propose to employ the unsteady RANS method with an advanced second-order moments-based turbulence model (so-called Reynolds stress model, RSM). The advantage of this model lies in its ability to automatically take into account exact production terms of the turbulent stresses (which need to be additionally modeled in the eddy-viscosity type of RANS model), as well as to predict turbulence anisotropy (in contrast to the assumption of turbulence isotropy as used in the eddy-viscosity turbulence models), which are important features of a flow in turbulent regime.

In the present study, we will first introduce a U-bend phantom that mimics the aorta with coarctation and produces numerous flow features observed in vivo. For the phantom, we will perform a detailed comparison between experiments (performed by 4D-flow MRI) and CFD simulations. In addition to the proposed RSM turbulence model, also two widely used eddy-viscosity-based turbulence models will be introduced. We will investigate levels of agreement between CFD and phantom experiments by focusing on the mean flow features and local distributions of the wall shear stress. Finally, we will perform a comparative assessment between CFD simulation (based on the turbulence model which performed best for the phantom study) and in vivo 4D-flow MRI for the patient-specific pulsating blood flow in CoA.

## Results

### Phantom

#### 4D-flow MRI flow rate

The volumetric flow rate was extracted from 11 different locations distributed evenly along the length of the phantom using CAAS MR Solutions v5.0 to test the performance of the MRI acquisition. We calculated the error between the set inlet volumetric flow $\hbox {Q}_{0}$ and the flow extracted at the different cut planes $\hbox {Q}_{i}$. The average error in flow rate was $0.25 \pm 2.11\%$.

#### Comparison of turbulence models

**Fig. 1:**
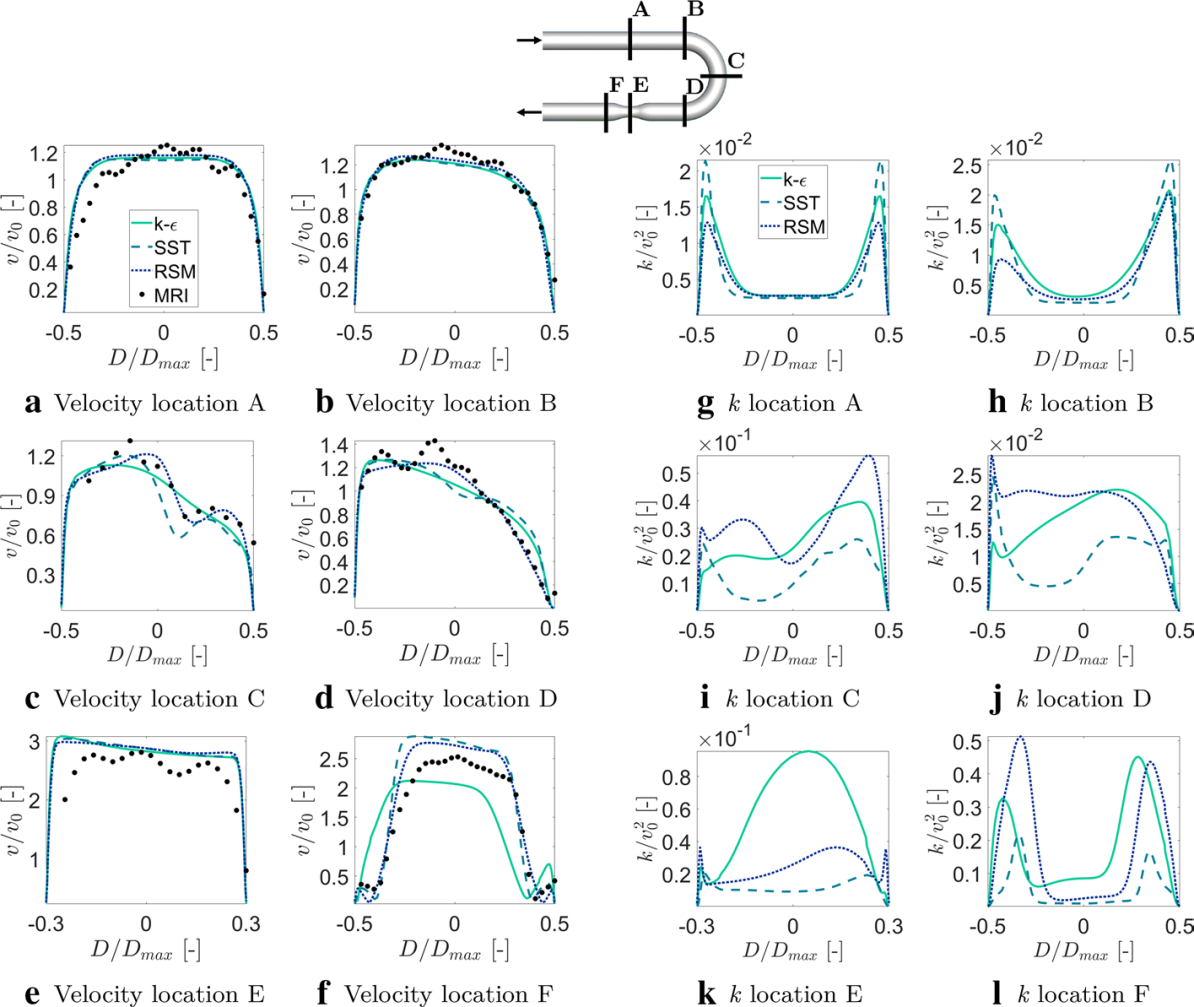
Comparison of measured (4D-flow MRI) and simulated (CFD) normalized velocity magnitude ($v/v_0$, where $v_0$ is the mean inlet velocity) and turbulent kinetic energy $k/v_0^2$ profiles at characteristic locations along the bend tube (**A**–**F**) (profiles extracted in the middle). The lines indicate various turbulence models: standard $k-\varepsilon$, SST (shear stress transport), and RSM (Reynolds stress model), respectively

Next, we moved towards a detailed comparison of the MRI and CFD simulations performed with various turbulence models by comparing the non-dimensional velocity ($v/v_0$, where $v_0$ is the mean inlet velocity) magnitude profiles at six characteristic locations: inlet (A), the start of the bend (B), middle of bend (C), end of the bend (D), middle of narrowing (E), and distal to narrowing (F), as shown in Fig. 1. The differences between the models emerge in the middle of the bend, i.e., at location C. This location is particularly sensitive due to the generation of the secondary flows (Dean vortices) and flow acceleration along the outer wall curvature. Additionally, at the post-stenotic location (location F), numerical simulations captured well the recirculation region, however, the $k-\varepsilon$ model underestimated the velocity magnitude in the center, whereas both SST and RSM models are showing a very good agreement with MRI in the wall vicinity, with a slight overprediction in the center.

The observed changes in the distributions of the velocity profiles for CFD with considered turbulence models are due to different predictions of turbulence levels. To illustrate this, we plot series of the profiles of the non-dimensional turbulent kinetic energy (${\mathrm{k/v}}_0^2$) at identical locations as previously analyzed for the velocity profiles (locations A–F), Fig. 1g–l. All turbulence models are giving similar profiles at inlet segment location (A), with almost identical values in the center and symmetrical peak values in the proximity of the wall and are in good agreement with the previously reported results [16]. The symmetrical distribution is, with elevated turbulence levels in the proximity of the outer wall, due to the presence of the bent (B–D). It is interesting to note that in the center of stenosis (location E), despite a big over-prediction of turbulent kinetic energy by the $k-\varepsilon$ model, resulting velocity magnitudes still agree very well due to the dominance of convective term in the momentum equation (due to a sudden flow acceleration). Finally, it can be seen that the post-stenotic region (location F) is characterized by the highest levels of turbulence caused by combined effects of a flow acceleration (in the center) and flow recirculation (in the wall proximity).

#### Voxel-to-voxel velocity and vorticity comparisons

**Fig. 2:**
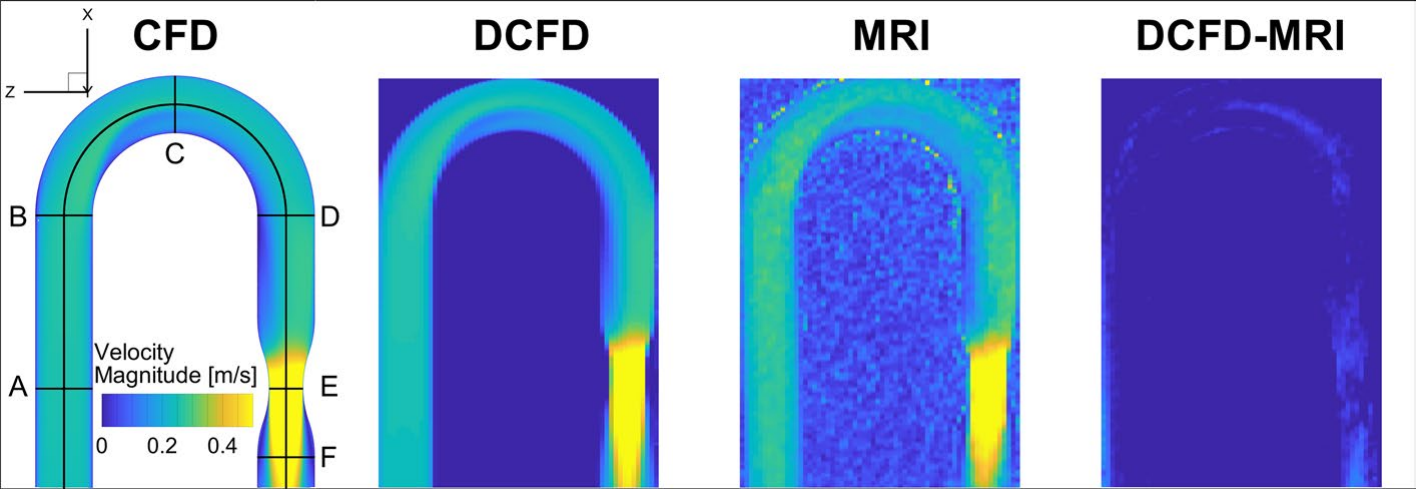
The contours of the velocity magnitude (|*v*|) in the central horizontal plane for CFD (original resolution), DCFD (downsized resolution), and MRI (original resolution), where the last contour indicates the absolute difference between DCFD and MRI

As the next step, we will compare in more detail (voxel-to-voxel) results of CFD (with the best performing turbulence model, RSM) against MRI. The contours of the velocity magnitude in the central horizontal cross-section ($\hbox {y} = 0$) are shown in Fig. 2. Here, we present the velocity magnitude distribution on original CFD resolution (CFD), downsized CFD resolution (DCFD, where downsizing is done to match original MRI resolution), original measurements (MRI), and the absolute difference between downsized CFD and MRI (DCFD-MRI), respectively. It can be seen that an overall good agreement is obtained between DCFD and MRI and that all most salient flow features are well captured with both techniques. The small deviations are located in the proximity of the walls (in the curved part) and central stenotic and post-stenotic regions.

**Fig. 3:**
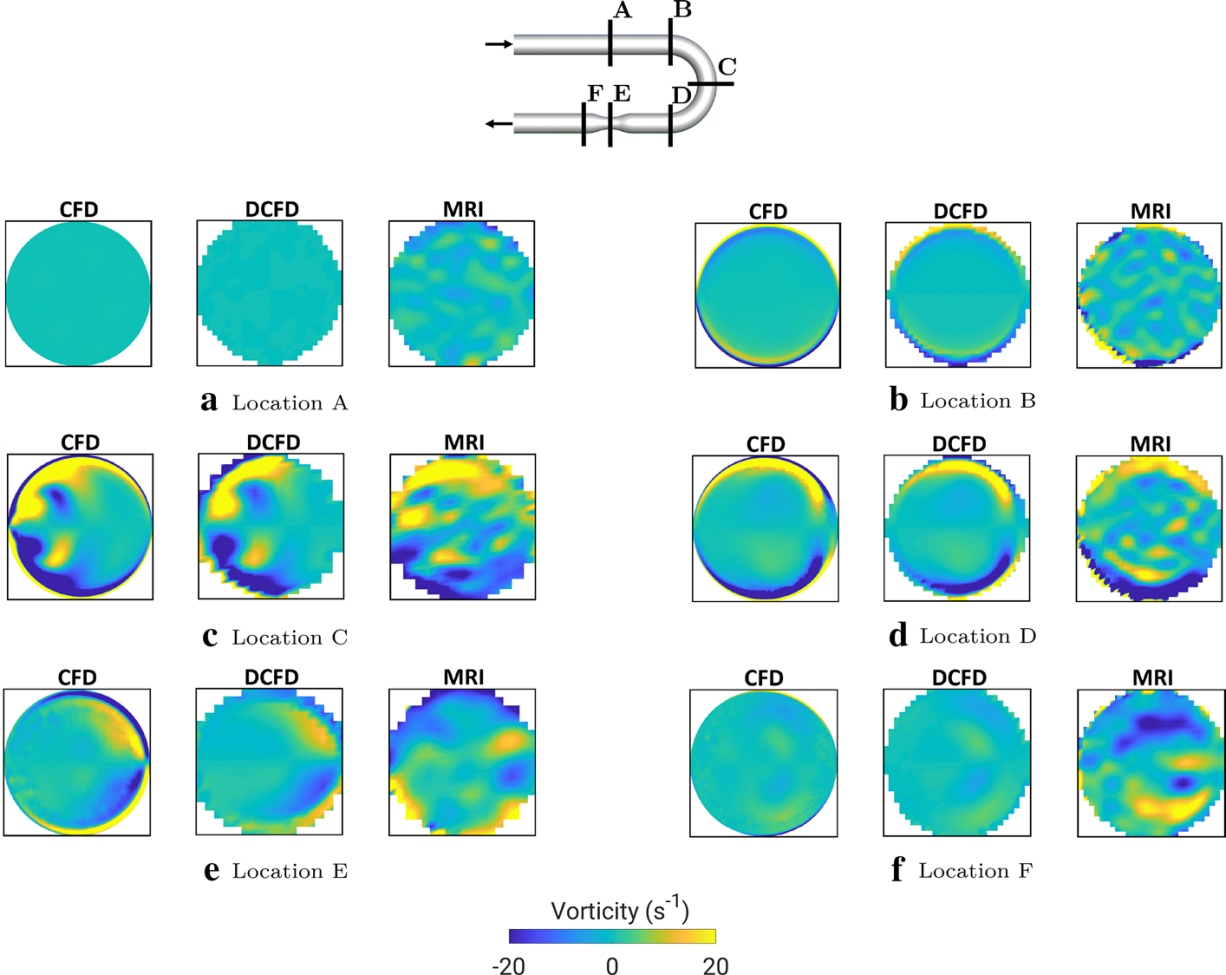
The contours of the out-of-plane vorticity component at selected cross-sections (**A**–**F**)—comparison of CFD (original spatial resolution), DCFD (downsized spatial resolution) and MRI

To compare secondary flow patterns, we plot contours of the out-of-plane vorticity component for all cases at characteristic selected cross-sections (A–F), Fig. 3. Note that the out-of-plane vorticity component (defined in here adopted coordinate system as: $\omega _x=\partial w/\partial y - \partial v/\partial z$) is a sensitive flow parameter since it captures gradients of both velocity components in the particular plane perpendicular to the flow direction. At the inlet segment location (A), there should not be yet any significant appearance of the secondary motions, as confirmed by CFD and DCFD results. The MRI contours show a more noisy distribution, but its levels are relatively small. By entering the bend curvature (location B), the vorticity starts to be generated. Here, due to limited spatial resolution, the MRI just partially captures some of secondary flow features. The agreement is much better at the most interesting location in the center of the bend (location C) where all cases captured well-detailed structure of the Dean vortices. The traces of Dean vortices are still visible at the end of the curved bend (location D), where a satisfactory agreement between CFD and MRI is obtained. A similar level of agreement is also obtained in the stenosis center (location E). Some visible deviations are observed in the post-stenotic region (location F), where the differences in vorticity magnitude are more pronounced.

#### Wall shear stress

**Fig. 4:**
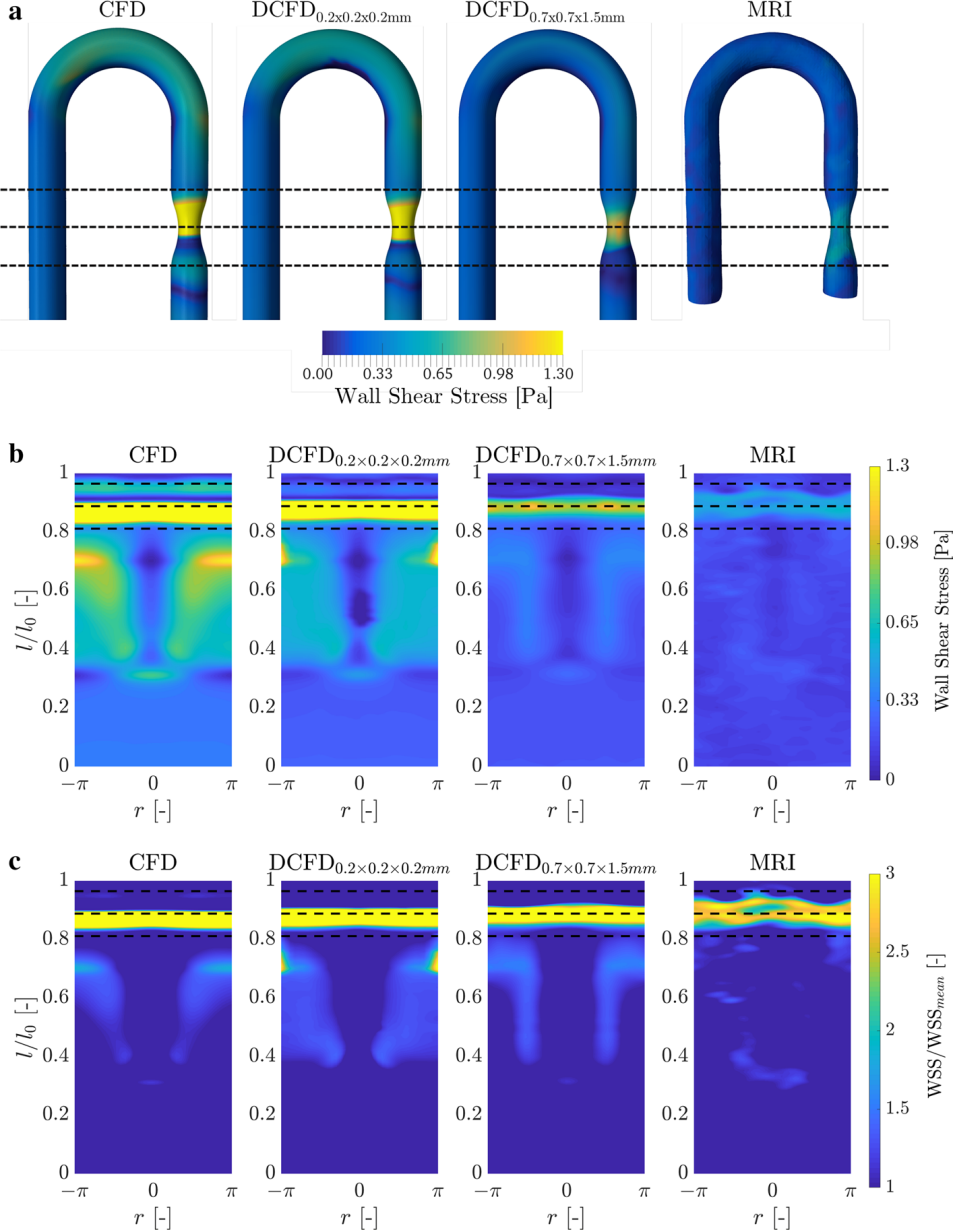
**a** The contours of the WSS at the phantom surface: CFD (original), DCFD (downsized) and MRI results; **b** the two-dimensional representation of the WSS at the phantom surface;** c** the two-dimensional map of the normalized WSS (WSS/$\hbox {WSS}_{\mathrm{mean}}$), where $\hbox {WSS}_{\mathrm{mean}}$ is a spatially averaged mean WSS calculated for each modality

**Fig. 5:**
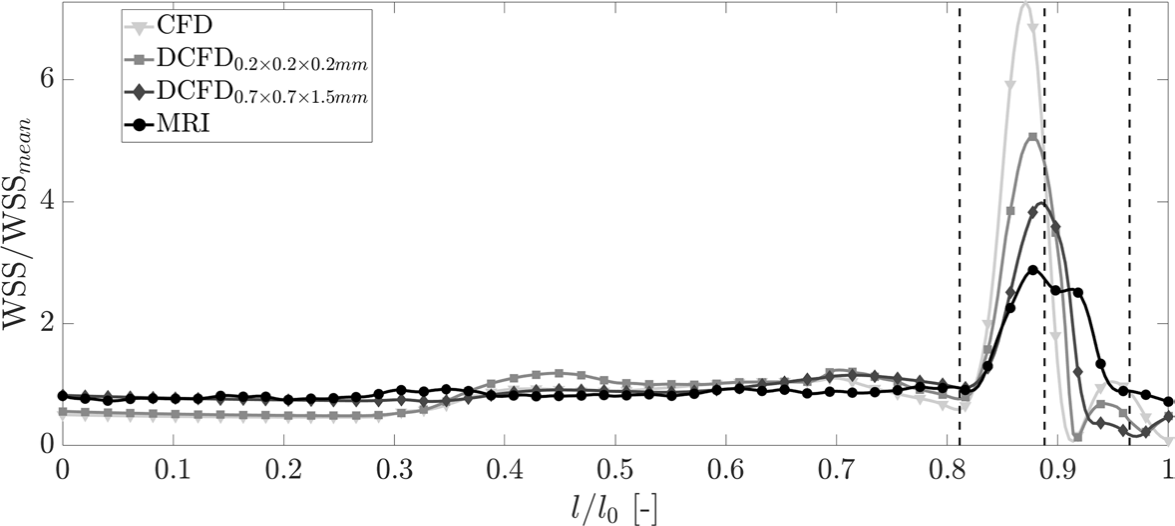
Profiles of the non-dimensional circumferentially averaged wall shear stress (WSS/$\hbox {WSS}_{\mathrm{mean}}$) obtained from original and downsized CFD results and MRI

The contours of the wall shear stress along the phantom walls for the original CFD (obtained with RSM turbulence model), downsized CFD results ($\hbox {DCFD}_{0.2\times 0.2\times 0.2}$ and $\hbox {DCFD}_{0.7\times 0.7\times 1.5\,\hbox {mm}^3}$), and original MRI are shown in Fig. 4a. To provide a complete distribution of the WSS along the phantom wall, we generated two-dimensional maps of WSS, where the horizontal coordinate represents the non-dimensional circumference (expressed in angles, $-\pi \le r \le +\pi$, where $r=0$ indicates the inner-curve and $r=\pm \pi$ indicate the outer phantom curve), while the vertical coordinate represents the non-dimensional enveloped arc-length of the phantom ($0\le l/l_0 \le 1$, where 0 and 1 correspond to the start and the end of the phantom, and $l_0$ is the centerline length), Fig. 4b. The non-dimensional WSS maps (WSS/$\hbox {WSS}_{\mathrm{mean}}$, where $\hbox {WSS}_{\mathrm{mean}}$ indicates the spatially averaged WSS over the entire phantom surface, given in Table 1) are shown in Fig. 4c. Finally, profiles of the circumferentially averaged non-dimensional WSS are shown in Fig. 5.

The agreement between CFD and MRI is good in the inlet leg of the phantom. The differences emerge in the bent and stenotic regions. Here, with the decreasing resolution, the absolute values of WSS decrease. It can be seen that all cases are predicting high values of WSS in the stenotic region (within the dashed lines), but lowering of the spatial resolution of the CFD results and MRI produced a slight shift of the location where WSS reached its maximum value.

**Table 1:** The maximal values of WSS during peak systole in the stenosis ($\hbox {WSS}_{{\mathrm{st}}}$), spatially averaged values of WSS during peak systole ($\hbox {WSS}_{{\mathrm{mean}}}$) and its standard deviation for the simplified phantom for MRI, $\hbox {DCFD}_{0.7\times 0.7\times 1.5 \,{\mathrm{mm}}^3}$, $\hbox {DCFD}_{0.2\times 0.2\times 0.2\,{\mathrm{mm}}^3}$, original CFD, and the peak shift (normalized by the inlet diameter $d_0$) in MRI and downsized CFD (with respect to the original CFD)

	$\hbox {WSS}_{{\mathrm{st}}}$ [Pa]	$\hbox {WSS}_{{\mathrm{mean}}}$ [Pa]	Standard deviation [Pa]	Peak shift$^{*}$ [−]
MRI	0.60	0.18	0.09	$0.39\textit{d}_{0}$
$\hbox {DCFD}_{0.7\times 0.7\times 1.5 \,{\mathrm{mm}}^3}$	1.10	0.25	0.17	$0.22\textit{d}_{0}$
$\hbox {DCFD}_{0.2\times 0.2\times 0.2 \,{\mathrm{mm}}^3}$	2.35	0.45	0.41	$0.11\textit{d}_{0}$
CFD	4.99	0.68	0.80	-

$^{*}$The shift is calculated with respect to the original resolution CFD

### Patient-specific CoA: in vivo MRI and CFD based on RSM turbulence model

**Fig. 6:**
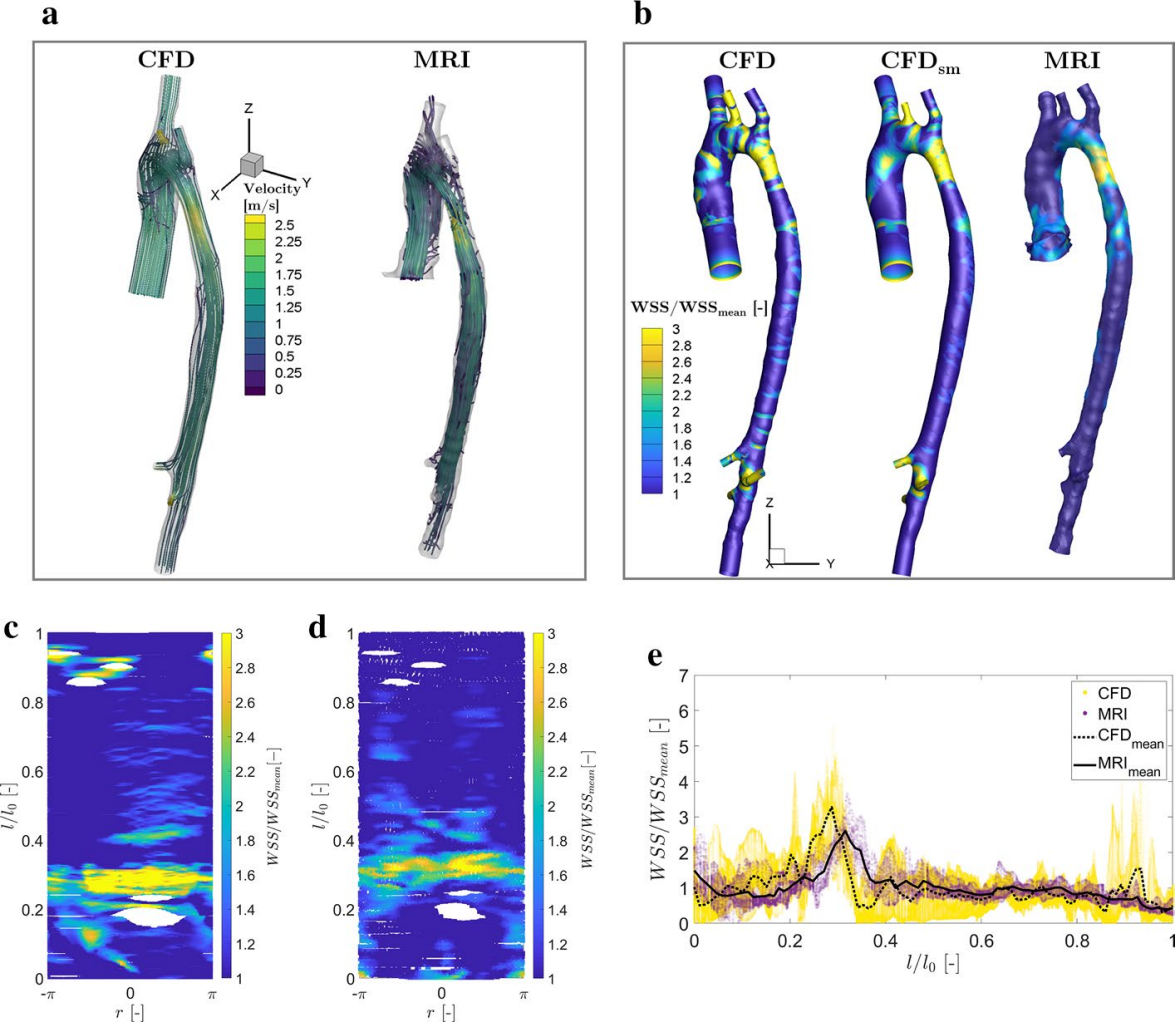
The patient-specific CoA case: the stream-traces colored by the velocity magnitude at the peak systole for CFD and 4D flow MRI (**a**); the contours of the non-dimensional WSS (WSS/$\hbox {WSS}_{\mathrm{mean}}$) at the aorta wall (**b**); the 2D map of WSS distribution for $\hbox {CFD}_{{\mathrm{sm}}}$ (**c**) and 4D-flow MRI (**d**); the scatter plots (symbols) and circumferentially averaged profiles (lines) of the non-dimensional WSS for 4D-flow MRI and $\hbox {CFD}_{{\mathrm{sm}}}$ (**e**)

**Fig. 7:**
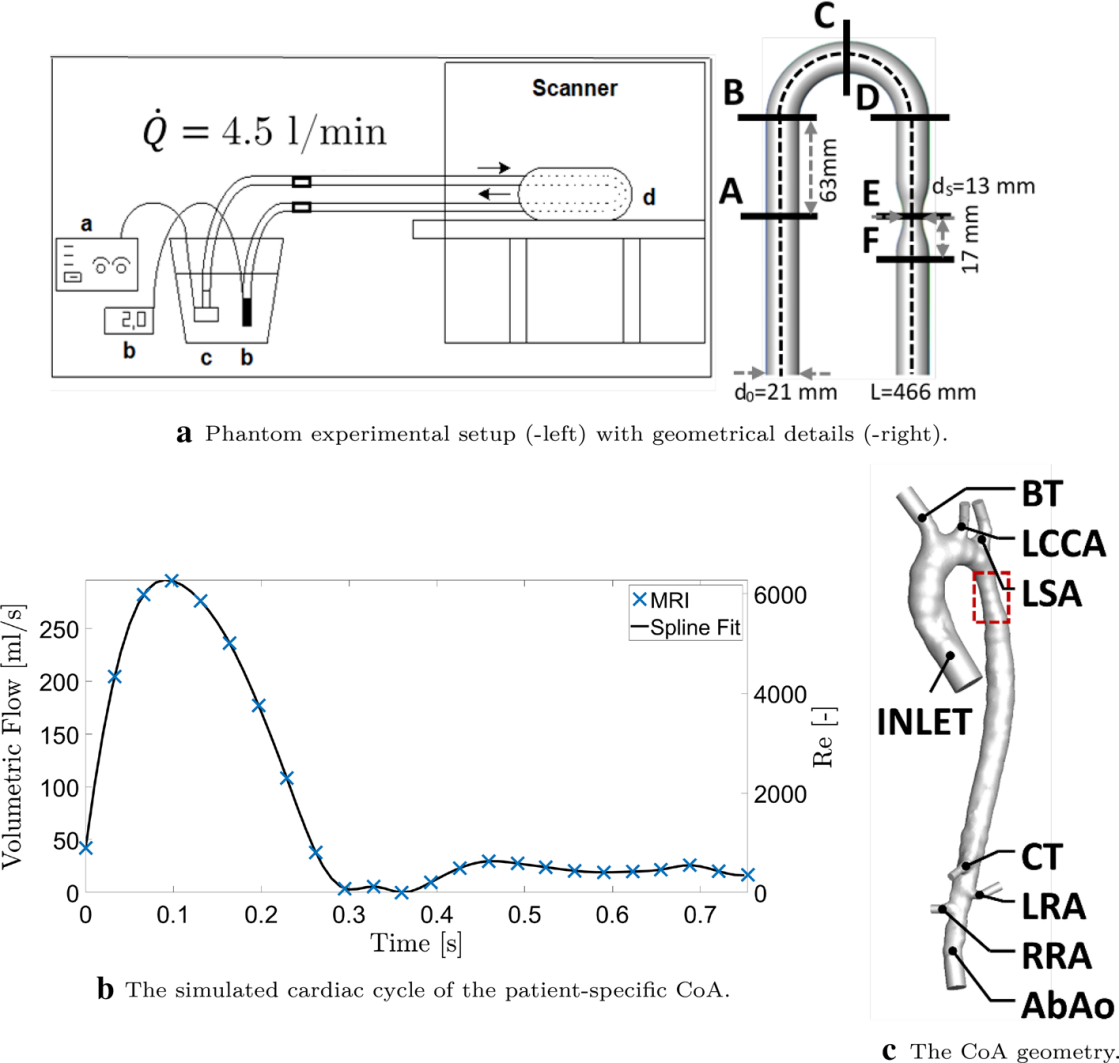
**a** Phantom experimental setup (-left) with: variable power source (**a**), flow indicator (**b**), submersible pump (**c**), flow phantom in the MRI scanner (**d**), and the phantom geometry (-right) represented with the main diameter $d_0$, stenotic diameter $d_S$, and total length of *L*;** b** the mass flow rate at the inlet of the patient-specific aorta with coarctation (CoA) from MRI and the fitted spline;** c** the patient-specific CoA with a highlighted position of narrowing (in red), inlet, and the branching arteries: brachiocephalic trunk (BT), left common carotid artery (LCCA), Left subclavian artery (LSA), celiac trunk (CT), left renal artery (LRA), right renal artery (RRA), and the rest of abdominal aorta (AbAo)

After demonstrating that CFD with RSM turbulence model was sufficient to predict the characteristic flow features in the phantom, we next moved to the patient-specific aorta geometry with coarctation, for which in vivo 4D-flow MRI measurements are available, Fig. 7c. The resulting flow pattern, presented in form of stream-traces colored by the velocity magnitude at the peak systole is shown in Fig. 6a. It can be seen that a good agreement between MRI and CFD is obtained in capturing important flow features: a strong helical pattern in the aortic arch, and a sudden flow acceleration in the coarctation. A summary of direct comparison between CFD and MRI in predicting the peak and spatially averaged (mean) values of the WSS is provided in Table 2. It can be seen that in vivo MRI underestimated the local values of WSS, similarly to our previous findings in the phantom geometry. The effect of the surface smoothing revealed relatively small differences between the rough and smoothed geometries. Note that present CFD results agree well with similar numerical studies reported in the literature, e.g., [17–19] Instead of focusing on the local differences in WSS from MRI and CFD in their absolute terms, we proceed with qualitative comparisons between simulations and experiments by identifying regions along aorta walls characterized by locally elevated or lowered values of WSS to their spatially averaged mean values (averaged over entire aorta wall). The contours of the non-dimensional WSS distribution (WSS/$\hbox {WSS}_{\mathrm{mean}}$) for CFD (both rough and smoothed geometry) and MRI are given in Fig. 6b. It can be seen that an overall good agreement is obtained, especially when considering the smoothed CFD and MRI distributions in the coarctation and the descending part of aorta. This is additionally illustrated by showing 2D maps of the local non-dimensional WSS, where the entire surface of aorta wall is mapped, Fig. 6c, d. The blank spaces in the mapped surfaces represent the branching arteries that were removed during the mapping procedure. Due to the proximity of the first two branching arteries (i.e., brachiocephalic trunk and left common carotid artery), they are merged on the mapped surface.

Finally, the scatter plots (symbols) and circumferentially averaged non-dimensional WSS profiles (lines) are shown in Fig. 6e. Similar to comparisons in the phantom geometry, qualitatively good agreement is obtained with distinct peak values in the coarctation. A shift in the location of the maximal WSS for the MRI is also observed. Note that larger peaks of WSS from CFD at $l/l_0=0.9$ locations are due to the secondary side branches of the aorta which are not properly resolved in MRI.

**Table 2:** The maximal values of WSS in the stenosis ($\hbox {WSS}_{{\mathrm{st}}}$), mean values of WSS ($\hbox {WSS}_{{\mathrm{mean}}}$) and its standard deviation of the patient-specific aortic coarctation (without branches) for MRI, CFD, smoothed CFD ($CFD_{sm}$), and the peak shift normalized by the inlet diameter $d_0$ in MRI with respect to $\hbox {CFD}_{{\mathrm{sm}}}$

	$\hbox {WSS}_{{\mathrm{st}}}$ [Pa]	$\hbox {WSS}_{{\mathrm{mean}}}$ [Pa]	Standard deviation [Pa]	Peak shift$^{*}$ [−]
MRI	7.73	2.12	0.97	0.52$d_0$
CFD	48.95	15.06	12.43	-
$\hbox {CFD}_{{\mathrm{sm}}}$	45.87	14.00	10.00	-

$^{*}$ The shift is calculated with respect to $\hbox {CFD}_{{\mathrm{sm}}}$

## Discussion

The present study investigated the flow and WSS for a specific aorta pathology—aortic coarctation—using a combined 4D-flow MRI and CFD techniques for simplified (phantom) and patient-specific geometry. For both studied geometries, the flow is in a fully (phantom) or a partially developed (CoA) turbulent regime. The importance of selected turbulence models for CFD is demonstrated by performing a comparative assessment between two types of eddy-viscosity models and RSM for the phantom configuration.

### Comparison of turbulence models with MRI

The differences in the cross-section averaged velocity magnitude profiles (at A–F locations) between MRI and CFD with the RSM model did not exceed 15% in the stenotic region and 7% in the rest of the phantom. In contrast, the maximum disagreement between CFD with $k-\varepsilon$ model and MRI was 45% for the post-stenotic region, while this disagreement was reaching 18% in the stenotic region for the SST model. The overall best performances of the RSM turbulence models can be explained in terms of the theoretical foundation behind this model. The exact treatment of production terms of the individual turbulent stress components plays a crucial importance in complex three-dimensional flows (e.g., curved part of the phantom followed by a stenotic region) as presented here. In comparison with the eddy-viscosity models, the RSM predicts well the secondary motions and captures well flow adaptation to sudden changes of the cross-sectional area [20]. This was also shown in terms of streamwise velocity and turbulent kinetic energy in a $60^{\circ }$ bend tube [21], where RSM performed best in comparison to other commonly used eddy-viscosity models. Especially for turbulent kinetic energy, while the eddy-viscosity models tend to under-predict the DNS-based values, RSM can capture the behavior better. We have shown this also in our results, where RSM-based turbulent kinetic energy showed slightly higher peaks in the curved part of the phantom. Based on this and direct comparison of performances of turbulence models with MRI measurements in the phantom, we conclude that the RSM turbulence model is the most suitable to properly capture the most important flow features. Additionally, in terms of computational efficiency, although a larger number of transport equations needs to be solved by the RSM turbulence model when compared to the eddy-viscosity-based models, its computational costs are still much smaller when compared to high-fidelity LES or DNS methods (i.e., ${{{\mathcal {O}}}} (10^2 - 10^3)$ faster, respectively), which makes it a good choice for the patient-specific clinical applications.

### Wall shear stress based on CFD and MRI

Two main points need to be addressed when comparing simulations (CFD) and experiments (MRI): (i) the absolute values of the WSS, and (ii) the local distributions of the WSS, respectively. Generally, we observed consistent lower values of WSS from MRI in comparison to CFD results. This can be explained in terms of the lower spatial resolution of MRI—especially in the proximity of the wall. The absolute values of the spatially averaged $\hbox {WSS}_{\mathrm{mean}}$ from CFD simulations for the phantom are $3.7\times$ (CFD), $2.5\times$ ($\hbox {DCFD}_{0.2\times 0.2\times 0.2 \,{\mathrm{mm}}^3}$), and $1.4\times$ ($\hbox {DCFD}_{0.7\times 0.7\times 1.5 \,{\mathrm{mm}}^3}$) higher than the MRI values, respectively. Similarly, the peak WSS in the stenotic region ($\hbox {WSS}_{\mathrm{st}}$) for CFD simulations are $8.3\times$ (CFD), $3.9\times$ ($\hbox {DCFD}_{0.2\times 0.2\times 0.2 \,{\mathrm{mm}}^3}$), and $1.83\times$ ($\hbox {DCFD}_{0.7\times 0.7\times 1.5 \,{\mathrm{mm}}^3}$) higher than the MRI values. Note that the stenotic region is the most sensitive one due to a sudden flow acceleration, and a reduction of the number of voxels (since the spatial resolution of MRI is fixed). In contrast, the CFD wall resolution in this region is increased since the identical number of control volumes as for the healthy segment is now distributed over the reduced area of stenotic cross-section. It can be seen that reduction of spatial resolution of CFD, lowers values of WSS. A similar systemic undersolving of WSS by MRI was also reported in [7].

In contrast to the absolute values of WSS, the local distributions of WSS calculated from CFD and measured by MRI exhibit more similarities. To illustrate this, we scaled the local WSS with the spatially averaged mean WSS ($\hbox {WSS}_{\mathrm{mean}}$) of each modality, as shown in Fig. 4c. This approach enables us to compare variations of WSS associated with locally elevated or suppressed distributions for the reference averaged value. The circumferentially averaged mean WSS profiles, shown in Fig. 5, show a good agreement except at the stenotic region. It can be seen that the reduction of spatial resolution of CFD reduced the peak values, but also introduced a shift of the peak location. Similar behavior was shown also in a related study of [22], but these findings were not addressed. This shift should be taken into account when analyzing cases where the exact location of the peak WSS is of importance.

### Effect of assumptions in CFD

The reliability and accuracy of CFD simulations in studying blood flow in the patient-specific conditions are directly connected to realistic representations of vessel geometry, as well as the imposed boundary conditions. The geometry representation can affect the simulations by not including all of the side branches [23] and due to the segmentation variability [24]. For the aortic coarctation studies, the choice of proper inlet and outlet side-branching boundary conditions was highlighted in [19] and [25], respectively. Finally, the assumption of rigid-wall in patient-specific simulations can lead to differences up to 30% [26].

Our approach to perform analysis for a simplified phantom is based on a step-by-step elimination process of specific contributions (e.g., exact wall geometry with all side branches, the exact specification of the inlet and outlet boundary conditions, wall elasticity) which make a fair comparison between CFD and MRI for the patient-specific cases difficult. By eliminating the effects of the above-mentioned limitations, we have achieved identical phantom working conditions for simulations and experiments. Despite achieving a good agreement between CFD and MRI for the mean velocity profiles at various cross-sections of the phantom, the local distribution of the WSS still exhibited significantly different values. A similar level of disagreement was also reported in the literature [17, 18]. Based on the above-presented arguments, we conclude that the major contributor to disagreement between MRI and CFD is due to limitations in spatial resolution of MRI in the proximity of the aortic wall.

The effect of pre-processing on the outcomes of simulations is also highlighted in the patient-specific CoA. For this case, a more drastic smoothing of the aortic wall resulted in a decrease in mean WSS of 7.3%, bringing the value closer to MRI. However, this decrease is still relatively small, comparing to almost an order-of-magnitude difference in WSS between MRI and CFD and both simulations resulted in very similar global distributions of WSS.

Our thorough comparison of blood flow and WSS based on MRI and CFD showed, that the blood flow, in general, agreed well for both techniques. However, the derived variables, like WSS, deviate much more. The reasons behind the deviation can be found on both sides. As demonstrated in the phantom, the underestimation of WSS is mostly due to MRI not being able to capture the steep gradients in a region with sudden flow acceleration. However, the boundary condition treatment and preparation of the geometry in CFD has also a big effect. Patient-specific CoA showed that the application of flat parabolic profile leads to discrepancies in the ascending aorta, and using arterial geometry without adequate smoothing slightly overestimates CFD-based WSS. Hence, when evaluating the WSS differences between MRI and CFD attention should be given to the limitation of both techniques.

### Clinical applications

While in this work we present the MRI-CFD coupling using 4D-flow MRI data, the technique can be coupled with different imaging techniques for the acquisition of the anatomy—e.g., MRA imaging. Nevertheless, PC-MRI at the inlet is still necessary for accurate definition of boundary conditions [19]. With this integrated CFD-MRI approach, where CFD is based on the advanced RSM turbulence model, it is possible to generate simulation results with high spatial resolution and a high level of accuracy within a few hours. This may lead to several interesting clinical applications of the image-based CFD framework for diseased arteries. Potential examples include pre- and post-operative follow-up for patients suffering from CoA [12]. Furthermore, by studying a wider population of patients, biomarkers for re-stenosis could be identified similarly as was done for femoropopliteal arteries [27], which could lead to a predictive method for potential complications connected to CoA.

The proposed approach is not only limited to coarctation or aorta. It can be easily applied also in other aortic diseases (e.g., aneurysm and dissection), or to study the blood flow in different parts of the cardiovascular system. Examples of these applications have already been tested, for example in cerebral aneurysms [28], stenosis of major arteries [29], and pulmonary arteries [30], and show very good promise.

Finally, it is important to touch upon the feasibility of using image-based CFD in the clinical application. As we showed with this study, the methods have a great potential to study the long-term effects of diseases or to model the progression and predict the outcomes of chronic vascular diseases. However, the state of the methods at the moment does not allow for implementation in acute decision-making. This is especially due to the fact, that the simulations and their preparation is time-consuming and requires expert knowledge. Most medical doctors do not have adequate training to perform such simulations and therefore, experts in computational fluid dynamics should be involved. Hence, until improvements in the automatizing the image-based CFD are made, for example by implementing mesh-less methods (e.g., Solid Particle Hydrodynamics [31]), the usage of MRI or other imaging methods for diagnostics of acute cases is necessary.

### Limitations

For the MRI measurements in the simplified phantom, the 4D-flow MRI with non-segmented gradient-echo and echo-planar imaging (EPI) acceleration has been used. In contrast to that, for in vivo patient-specific CoA, the 4D-flow MRI with segmented gradient-echo without EPI has been applied. As result, some minor differences in accuracy in velocity quantitation may be present between these two experiments. Additionally, water was used as a working fluid for the flow in the phantom instead of blood mimicking fluid with non-Newtonian viscosity, e.g., as proposed by Cheng et. al. [32]. Use of such a fluid would represent the blood flow more adequately [32], however, since the shear rate in the aorta was relatively high the non-Newtonian effects should be minimal. The CFD simulations of the patient-specific CoA have been performed with the rigid walls assumption. The dynamic movement of the aorta is present in vivo 4D-flow MRI, which can produce differences in local distributions of WSS along the arterial wall. To circumvent the effects of the dynamic movement of the aorta during the cardiac cycle, the simplified phantom geometry has been considered too. Despite the relatively high Reynolds number of flow in the patient-specific aorta, some local non-Newtonian effects can take place, which is currently not taken into account in CFD simulations. We have considered a single case of the patient-specific CoA and have performed a comparative assessment of 4D-flow MRI and CFD-RSM as a first proof-of-concept. This study can be easily extended with a larger number of patient-specific aorta conditions.

## Conclusions

With this study, we showed that MRI-based CFD simulations are a good alternative tool to use in studying the blood flow in CoA. Using MRI and MRI-based simulations, we assessed the blood flow in a phantom representing a simplified CoA and in a patient-specific aorta with coarctation.

Due to the narrowing and relatively high Re, the flow in the phantom was of turbulent nature. Because of this, a choice of turbulent model had to be made. We have compared $k-\epsilon$, SST, and RSM. The differences between the turbulence model arise after the bend—where the flow gets more complex. The lower-order CFD models ($k-\epsilon$, SST) cannot accurately model the secondary flow motion that naturally appears in these types of geometries as is shown in the results obtained from MRI. However, the RSM model can predict these motions as was shown for both velocity magnitude as well as the out-of-plane vorticity. Thanks to using the phantom, where the boundary conditions between MRI and CFD are identical, we were able to accurately study WSS, an important parameter that is often regarded as a bio-marker for CoA. We showed that WSS based on MRI is approximately four-times lower than WSS based on CFD, however, agrees well in terms of the local distribution.

Finally, we have applied MRI-CFD coupling on patient-specific CoA to demonstrate usability of the technique for clinical applications. The agreement between 4D-flow MRI and CFD was good in terms of velocity. For WSS, simulations showed again higher values that MRI, however, the local regions of high/low WSS agree well between the different techniques. However, the simulations bring several advantages, like higher resolution and better prediction of the absolute values of WSS. This shows, that image-based simulations are a good technique to asses the state of this pathology.

## Methods

### Studied cases

#### Phantom

The phantom for this study was a 3D-printed computer-aided design object, mimicking the simplified aorta with coarctation. It represents a $180^{\circ }$ bend structure in which obstruction is present in the distal leg, as shown in Fig. 7a. The three-dimensional U-tube phantom is fabricated from Duraform Flex material with characteristic stiffness of a shore hardness scale of A75, manufactured by Materialise. The phantom can be considered as a rigid structure. To allow magnetic resonance imaging, the 3D U-tube phantom is placed inside a 10 l jerry can, which is filled with gelatin. Gelatin is used instead of water to prevent movement of the liquid due to the sound produced by the MRI system. The exact composition of the gelatin is 9.9 l water, 600 g gelatin, 100 ml paraben, and 1.5 ml Gadovist. The jerry can has two connectors to allow connection with the tubes through which the liquid will be pumped. A constant throughput is provided using a pump connected to the inlet tube with a prescribed flow of $\dot{Q_0}=4.5$ l/min. Additional morphometric and flow characteristics of the studied phantom can be found in Table 3.

**Table 3:** Morphometric, numerical mesh, and flow characteristics (showing the estimated thickness of boundary layer, Womersley number - Wo, inlet Reynolds number - $\hbox {Re}_{0}$, and stenotic Reynolds number - $\hbox {Re}_{{\mathrm{st}}}$) for the phantom and the patient with coarctation (CoA). Note that $\delta _1$ and $\delta _2$ are the estimated transient boundary layer thickness ($\delta _1=\sqrt{\nu /\omega }$) and averaged boundary layer thickness ($\delta _2=\sqrt{\nu D_0/U}$), respectively [33]

	Phantom	Patient with CoA
**Morphometric characteristics**		
$\hbox {D}_{0}$ [cm]	2.10	1.82
$\hbox {D}_{\mathrm{st}}$/$\hbox {D}_{0}$ [−]	0.62	0.60
**Mesh details**		
Bulk max. element size [mm]	0.80	0.75
$1^{{\mathrm{st}}}$ prism element thickness [mm]	0.05	0.05
Exponential growth factor	1.20	1.20
Number of prism layers	10	10
**Flow characteristics**		
$\delta _1$ [mm]	–	0.06
$\delta _2$ [mm]	0.32	0.23
$\hbox {Re}_{0}$ [−]	4,539	6,276
$\hbox {Re}_{st}$ [−]	7,332	11,425
Wo [−]	–	28.87

#### Patient-specific aorta with coarctation

This study protocol was approved by the Medical Ethics Committee of the Leiden University Medical Center (P14.095), and informed consent was signed by both parents/guardians of the subject. The patient with CoA included in this study was female, 14.5 years old, 164 cm, 51,9 kg, and had a tricuspid aortic valve. The final geometry includes both thoracic and abdominal aorta (AbAo) with six branching arteries: brachiocephalic trunk (BT), left common carotid artery (LCCA), left subclavian artery (LSA), celiac trunk (CT), and left/right renal artery (L/RRA) (visualized in Fig. 7c. Additional morphometric and flow characteristics of the studied CoA can be found in Table 3.

### Magnetic resonance imaging

For both, the phantom and patient-specific CoA, 4D-flow MRI was performed on a 3T MRI system (Ingenia, Philips Healthcare, Best, The Netherlands). For the patient-specific CoA, aortic 4D-flow MRI was performed using a hemidiaphragm respiratory navigator with retrospective electrocardiogram gating without echo-planar imaging. Additional file 1 about the MRI sequences can be found in Table 4.

**Table 4:** Details of MRI sequence for phantom and patient with aortic coarctation

	Phantom	Patient-specific CoA
Heart rate [bpm]	60 (simulated)	80
Velocity encoding [cm/s]	70	
Echo time [ms]	5.5	2.3
Repetition time [ms]	11	4.1
Reconstructed phases [−]	20	24
Flip angle [$^{\circ }$]	7	10
Field of view [$\hbox {mm}^{3}$]	–	$350\times 350 \times 52.5$
Acquired spatial resolution [$\hbox {mm}^{3}$]	$1.5 \times 1.5 \times 1.5$	$2.5 \times 2.5\times 2.5$
Reconstructed spatial resolution [$\hbox {mm}^{3}$]	$0.7\times 0.7\times 1.5$	$1.5 \times 1.5\times 2.5$
Echo-planar imaging factor (anterior–posterior direction)	5	-
Sense factor (anterior–posterior direction)	2	2
Acquisition time (without respiratory compensation) [min]	–	5.8

The acquired 4D-flow MRI data sets were afterward analyzed using CAAS MR Solutions v5.0 (Pie Medical Imaging BV, Maastricht, The Netherlands). The analysis is similar for both phantom and patient-specific CoA and the most important differences are highlighted. In both, the analysis was initialized by manual placement of starting and ending points. For the phantom, the two points were placed at the same level in the opposite legs, whereas for the patient-specific CoA, the starting point was placed in the aortic root, and the ending points were placed in the abdominal aorta and the six branching arteries. A phase-specific 3D volume was automatically segmented for the specific phase and copied to all phases. The 3D segmentation uses a deformable model algorithm that recursively optimizes the location of the surface towards the vessel luminal boundary based on image gradients, extracted from the appropriate phase within the 4D-flow MRI data, while simultaneously maintaining local smoothness of the 3D segmented surface, [34]. Manual delineation of the vessel lumen boundary was applied with the available adaptation tool from the software in case of segmentation incorrectness for the patient-specific CoA.

### CFD model

#### Numerical mesh

To perform CFD simulations of the patient-specific aorta geometry (obtained from 4D-flow MRI segmentation using CAAS MR Solutions v5.0), we first used Vascular Modeling Toolkit for the geometry reconstruction [35]. To check the sensitivity of CFD results on the imposed levels of geometry smoothing, we have used the rough ($\hbox {CFD}_{\mathrm{rough}}$) (i.e., by using a Taubin filter with 100 iterations and passband settings of 0.4), and the smoothed geometry ($\hbox {CFD}_{\mathrm{smooth}}$) (i.e., by using a Taubin filter with 100 iterations and passband settings of 0.01). For both geometries, cylindrical extensions were added at all outlets with a length of $1.5d_0$ (where $d_0$ is the diameter of the individual blood vessel where the outlet is located). For both phantom and patient-specific aorta simulations, we have employed a hybrid numerical mesh containing prismatic elements in the proximity of the wall (to properly resolve characteristic boundary layers), while the tetrahedrons were used in the central part of the domain. Details about the mesh sizing are shown in Table 3.

We have performed a mesh-independency study and the final numerical mesh for the phantom case was approximately 14 million control volumes, while approximately 7 million control volumes were used for the aorta case. Note that the larger number of control volumes for the phantom geometry was due to the addition of a segment with a length of $10 d_0$ to have a proper capture of the post-stenotic flow region (not shown in Fig. 7a).

#### Governing equations

Since we are dealing with a fully (the phantom case) or a partially (the aorta case) developed turbulent flow regimes, we adopt an unsteady RANS approach to model turbulence. We apply three different classes of turbulence models: two based on the eddy-viscosity concept (standard $k-\epsilon$ and shear-stress transport (SST) $k-\omega$ model), and an advanced turbulence model based on solving a complete set of the turbulent stress components (the full RSM). Despite its superior theoretical foundation when compared to the eddy-viscosity turbulence models [20], applications of the RSM model are very scarce in bio-medical flow applications. Here we propose the use of the RSM as an alternative to a high-fidelity DNS or LES approaches. The following set of the governing equations is introduced for the above-mentioned turbulence models:

- the standard eddy-viscosity $k-\epsilon$ model with enhanced wall treatment [36]: PDEs for ($U_i - p - k - \varepsilon$)
- the low-Reynolds shear stress transport (SST) $k-\omega$ model [37]: PDEs for ($U_i - p - k - \omega$)
- the Reynolds stress model (RSM) with the linear pressure strain term and enhanced wall treatment [38]: PDEs for ($U_i - p - \overline{u_i u_j} - \varepsilon$)

where $U_i$ is the velocity vector, *p* is the pressure, *k* is turbulent kinetic energy, $\varepsilon$ is dissipation rate, $\omega$ is turbulent frequency, and $\overline{u_i u_j}$ is turbulent stress tensor.

#### Boundary and initial conditions

For the phantom, the imposed volumetric flow rate of 4.5 l/min is identical to the experimental conditions and corresponds to the inlet Reynolds number of $Re=4539$ ($Re=V_0\cdot D_0/\nu$). For the patient-specific aorta, the time-dependent inlet conditions are matched with MRI measurements during the entire cardiac cycle. We have extracted the measured volumetric flow rate at the inlet plane ($Q_0$), and converted it to the characteristic mass flow rate (${\dot{m}}=Q_0 \cdot \rho _{\mathrm{blood}}$). The mass flow rates were fitted with a smooth spline with piecewise polynomial (with a smoothing parameter $p=0.99999947$ and $R^2=0.9995$), Fig. 7b, which gives the following range of the inlet Reynolds number, $0 \le Re \le 6276$, and corresponding Womersley number of $Wo=D_0 \left( \omega _f/\nu \right) ^{1/2} = 29$. In total, we have simulated five cardiac cycles, to obtain results without the influence of initial conditions. Only the last cycle was used for the analysis. For all turbulence parameters, the uniform inlet values were imposed with the following specifications: the intensity of turbulence of 5%, the ratio of turbulent and molecular viscosity ($\mu _t/\mu$) of 10, the isotropic assumption of normal turbulent stress components ($\overline{u_i u_i}=2/3 k$), and zero values of the turbulent shear stress components ($\overline{u_i u_j}=0$). At outlets, a zero diffusion flux was imposed for all transport variables. For the patient-specific aorta, a pre-defined fixed (MRI-based) percentage of the inlet flow rate was prescribed. The no-slip velocity boundary condition was imposed at the walls of blood vessels, and the model was assumed to be rigid.

#### Physical properties and simulation setup

For the phantom, water was used as a working fluid ($\rho =998\, \hbox {kg}/\hbox {m}^3$, $\mu =1.003\,\hbox {mPa}\cdot \hbox {s}$). For the aorta, the real blood properties were assumed for the simulations ($\rho =1060\,\hbox {kg}/\hbox {m}^3$, $\mu =3.5\,\hbox {mPa}\cdot \hbox {s}$). It was previously demonstrated that the assumption of constant blood viscosity is adequate for aortic blood simulations [39]. The simulations were performed using Ansys Fluent 19.1 (Ansys Inc., Canonsburg, Pennsylvania, USA) with the following simulation settings:

- Solver—pressure based
- Pressure–velocity coupling—SIMPLE
- Spatial discretization
  - Gradient-least-squares cell-based
  - Pressure—second order
  - Momentum—second-order upwind
  - Turbulence variables—second-order upwind
- Temporal discretization (CoA case)
  - Fully implicit second-order scheme
  - Time step $$\Delta t = 0.0005$$s
- Residuals (all)- $$10^{-5}$$

### Analysis

#### Downsizing and mapping

For additional analysis and voxel-to-voxel comparison, the phantom CFD velocity data were down-sampled by applying a bilinear interpolation on two different equidistant meshes (DCFD):

- $\hbox {DCFD}_{0.7\times 0.7 \times 1.5}$: voxel resolution $0.7\times 0.7\times 1.5\,\hbox {mm}^3$ (identical to MRI)
- $$\hbox {DCFD}_{0.2\times 0.2 \times 0.2}$$: voxel resolution $$0.2\times 0.2\times 0.2\,\hbox {mm}^3$$.

Both downsized CFD velocity fields were analyzed using CAAS MR Solutions v5.0 (Pie Medical Imaging BV, Maastricht, The Netherlands) to obtain WSS. Additionally, the WSS distribution along the vessel walls was mapped on a 2D surface where the horizontal axis indicates the non-dimensional radial distance from the vessel centerline ($-\pi \le r \le +\pi$), and the vertical axis indicates the non-dimensional arc-length of the vessel ($0 \le l/l_0 \le 1$). This mapping was done by an originally developed in-house tool in Matlab R2019a (MathWorks, Inc., Natick, Massachusetts, U.S.A.). This approach has provided an easy and objective comparison between different results.

#### Vorticity calculation

Vorticity (${\varvec{\omega }}$) was calculated from MRI-based velocity components using Matlab R2019a (MathWorks, Inc., Natick, Massachusetts, U.S.A.) as

1 $$\begin{aligned} {\varvec{\omega }}=\nabla \times {\varvec{u}}, \end{aligned}$$

where ${\varvec{u}}$ is the velocity vector. In the numerical procedure, the partial derivatives were calculated using a central differencing scheme for the interior points and a single-sided (forward) difference scheme for the edges.

#### Wall shear stress calculation

The WSS for MRI and the downsized CFD data sets were calculated based on the extracted velocity profile perpendicular to the phase-specific segmented 3D surface using CAAS MR Solutions v5.0. After factorizing the velocity profile into its component parallel to the lumen wall, WSS was computed by the first derivative of a quadratic approximation of that velocity profile at the location of the lumen wall as

2 $$\begin{aligned} \tau _w=\mu \frac{{\partial }U_{||}}{{\partial }n}\Bigg |_{\mathrm{wall}} \end{aligned}$$

where $U_{||}$ is the wall-parallel velocity component and *n* is the wall-normal direction. For the CFD simulations involving turbulence models, the wall shear stress is directly available from calculations of the wall-parallel velocity component based on the enhanced wall treatment.

## Supplementary Information

**Additional file 1.** Mesh Dependency Study.** Table S1.** Mesh dependency analysis for phantom and patient, with wall shear stress for three different meshes (fine - 1, medium - 2, coarse - 3), refinement ratio* r*, Richardson extrapolation (fh=0), Grid Convergence Index (GCI1,2 - fine-medium, GCI2,3 - medium-coarse), and the test whether the studied variables lie in the asymptotic range.** Figure S2.** Tetrahedral mesh with details of inlet and stenosed region for phantom (a) and patient-specific aorta (b).

## Data Availability

The datasets used and/or analyzed during the current study are available from the corresponding author on reasonable request.
