# Supplementary material for: Assessment of turbulent blood flow and wall shear stress in aortic coarctation using image-based simulations
Source: Biomed Eng Online. 2021 Aug 21;20:84. doi: 10.1186/s12938-021-00921-4 (PMC8379896; doi:10.1186/s12938-021-00921-4)
Supplement: Supplementary file 1 — Additional file 1. Mesh Dependency Study. Table S1. Mesh dependency analysis for phantom and patient, with wall shear stress for three different meshes (fine - 1, medium - 2, coarse - 3), refinement ratio r, Richardson extrapolation (fh=0), Grid Convergence Index (GCI1,2 - fine-medium, GCI2,3 - medium-coarse), and the test whether the studied variables lie in the asymptotic range. Figure S2. Tetrahedral mesh with details of inlet and stenosed region for phantom (a) and patient-specific aorta (b). [file 12938_2021_921_MOESM1_ESM.docx]

# Additional file 1

## Mesh Dependency Study

In Table [1](#_bookmark62) we give an overview of the Grid Convergence Index (GCI) analysis for the two studied cases. We have based the analysis on mean WSS since WSS was the main study objective in this research. Three meshes, ith tetrahedral elements and prism layer close to the wall were created for each of the cases:

- Fine (mesh 1) - phantom (14.1 million), patient CoA (9.3 million)
- Medium (mesh 2) - phantom (7.0 million), patient CoA (4.7 million)
- Coarse (mesh 3) - phantom (3.6 million), patient CoA (2.3 million).

Both the Richardson extrapolation and the test whether the studied parameter lies in the asymptotic range show that the medium mesh is sufficient. Based on the analysis, we have proceeded with mesh 2 (medium), for all presented results.

The details of the final numerical meshes that were used for the two studied geometries are shown in [Fig. 1.](#_bookmark61)

Table 1: Mesh dependency analysis for phantom and patient, with wall shear stress for three different meshes (fine - 1, medium - 2, coarse - 3), refinement ratio r, Richardson extrapolation (fh=0), Grid Convergence Index (GCI1,2 - fine-medium, GCI2,3 - medium-coarse), and the test whether the studied variables lie in the asymptotic range


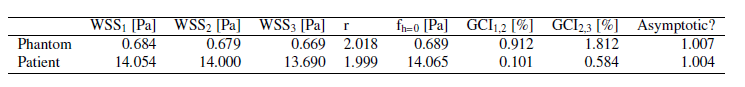


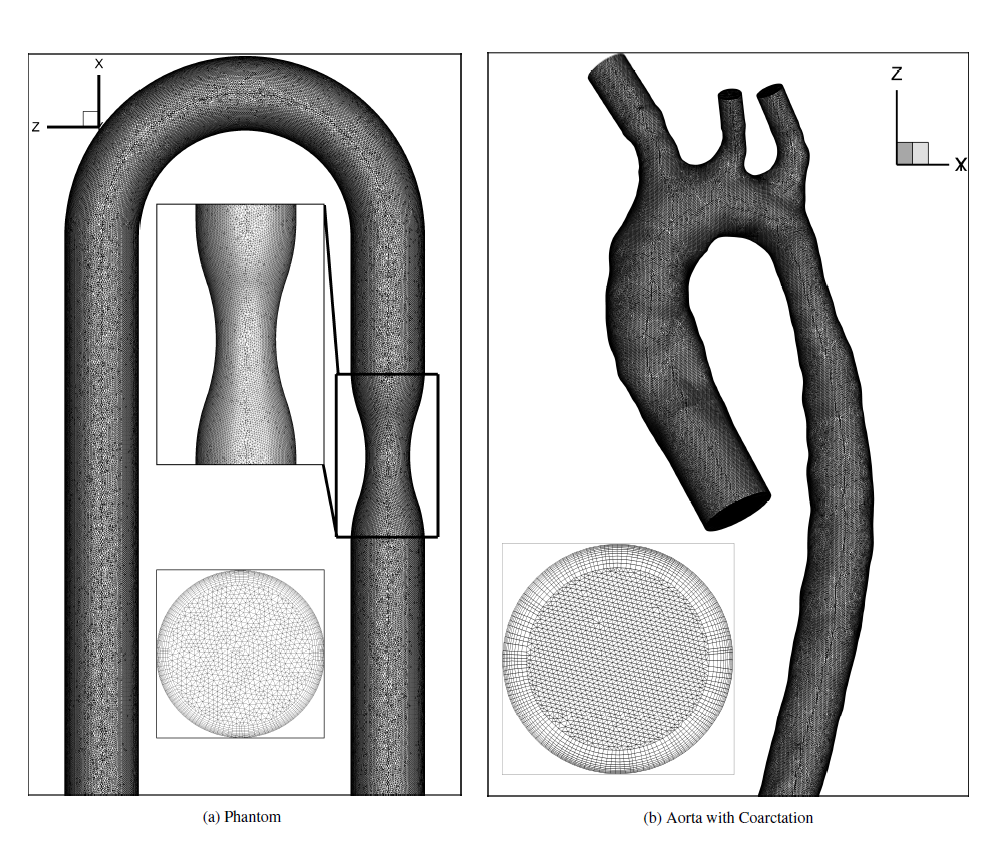


Figure 2: Tetrahedral mesh with details of inlet and stenosed region for phantom (a) and patient-specific aorta (b)
